# Visual dysfunction is a better predictor than retinal thickness for dementia in Parkinson’s disease

**DOI:** 10.1136/jnnp-2023-331083

**Published:** 2023-04-20

**Authors:** Naomi Hannaway, Angeliki Zarkali, Louise-Ann Leyland, Fion Bremner, Jennifer M Nicholas, Siegfried K Wagner, Matthew Roig, Pearse A Keane, Ahmed Toosy, Jeremy Chataway, Rimona Sharon Weil

**Affiliations:** 1 Dementia Research Centre, Institute of Neurology, University College London, London, UK; 2 National Hospital for Neurology and Neurosurgery, University College London Hospitals, London, UK; 3 Department of Medical Statistics, London School of Hygiene and Tropical Medicine, London, UK; 4 Moorfields Eye Hospital, London, UK; 5 UCL Queen Square Institute of Neurology, London, UK; 6 Institute of Ophthalmology, University College London, London, UK; 7 Queen Square Multiple Sclerosis Centre, Department of Neuroinflammation, UCL Queen Square Institute of Neurology, University College London, London, UK; 8 National Institute for Health Research, University College London Hospitals Biomedical Research Centre, London, UK; 9 MRC CTU at UCL, Institute of Clinical Trials and Methodology, University College London, London, UK; 10 Movement Disorders Centre, University College London, London, UK; 11 The Wellcome Centre for Human Neuroimaging, Institute of Neurology, University College London, London, UK

**Keywords:** vision, dementia, Parkinson's disease, cognition

## Abstract

**Background:**

Dementia is a common and devastating symptom of Parkinson’s disease (PD). Visual function and retinal structure are both emerging as potentially predictive for dementia in Parkinson’s but lack longitudinal evidence.

**Methods:**

We prospectively examined higher order vision (skew tolerance and biological motion) and retinal thickness (spectral domain optical coherence tomography) in 100 people with PD and 29 controls, with longitudinal cognitive assessments at baseline, 18 months and 36 months. We examined whether visual and retinal baseline measures predicted longitudinal cognitive scores using linear mixed effects models and whether they predicted onset of dementia, death and frailty using time-to-outcome methods.

**Results:**

Patients with PD with poorer baseline visual performance scored lower on a composite cognitive score (β=0.178, SE=0.05, p=0.0005) and showed greater decreases in cognition over time (β=0.024, SE=0.001, p=0.013). Poorer visual performance also predicted greater probability of dementia (χ² (1)=5.2, p=0.022) and poor outcomes (χ² (1) =10.0, p=0.002). Baseline retinal thickness of the ganglion cell–inner plexiform layer did not predict cognitive scores or change in cognition with time in PD (β=−0.013, SE=0.080, p=0.87; β=0.024, SE=0.001, p=0.12).

**Conclusions:**

In our deeply phenotyped longitudinal cohort, visual dysfunction predicted dementia and poor outcomes in PD. Conversely, retinal thickness had less power to predict dementia. This supports mechanistic models for Parkinson’s dementia progression with onset in cortical structures and shows potential for visual tests to enable stratification for clinical trials.

**WHAT IS ALREADY KNOWN ON THIS TOPIC**

- Visual dysfunction is emerging as a predictor for cognitive decline in Parkinson’s disease. However, it is not yet clear whether higher order visual impairment or ophthalmic factors especially retinal measures are better predictors of Parkinson’s dementia.

**WHAT THIS STUDY ADDS**

- We showed that higher order visual dysfunction is a more robust predictor of dementia and poor outcomes in Parkinson’s disease than retinal thickness, in 100 patients followed-up over 3 years.

**HOW THIS STUDY MIGHT AFFECT RESEARCH, PRACTICE OR POLICY**

- This study confirms that higher order visual tests could be useful to stratify patients to enrich clinical trials and as predictive measures in the clinic; but that structural retinal measures are less likely to be effective in this way.

## Introduction

Dementia affects up to 50% of people with Parkinson’s disease (PD) within 10 years of diagnosis,^1^ a rate six times higher than in the general population^2^ and with double the financial burden of other dementias.^3^ Heterogeneity in timing and severity of cognitive involvement is a major challenge in understanding PD dementia. Predicting who is most likely to develop dementia is increasingly important with the emergence of disease-modifying treatments.^4^ This would enable patients most at-risk to be targeted early for intervention and to reduce variability in clinical trials.

Visual dysfunction is well recognised in PD, with loss of spatial navigation, line orientation, visual rotation and facial recognition (reviewed in Weil *et al*).^5^ Retinal changes are also observed, with decreased retinal dopamine concentration^6^ and postmortem alterations in retinal structure.^7^ Inner retinal layers, especially the inner plexiform layer (IPL), are most affected, with phosphorylated alpha-synuclein found within the IPL at postmortem.^7^ This may relate to the location of dopaminergic amacrine cells, which are seen within this layer in non-human studies.^8^ Retinal structure can be measured non-invasively using optical coherence tomography (OCT) with several studies showing thinning in the IPL and ganglion cell layer (GCL) in PD.^9 10^

Recently, converging evidence has revealed that visual deficits are predictive for dementia in PD.^5^ Patients with impaired colour vision,^11^ pentagon copying^1^ and other higher order visual changes have a greater risk of developing dementia.^1 11–14^ This relationship has been confirmed in large-scale population studies; patients with PD with visual impairment showed increased risk of dementia, falls and death.^15 16^ Neuroimaging also supports these observations: occipital hypometabolism is seen at baseline in patient with PD who progress to dementia after follow-up,^17^ and patients with baseline visuoperceptual deficits show more widespread and diffuse white matter damage over time than patients with intact visuoperceptual function.^13^

Evidence that retinal changes may relate to cognitive dysfunction in PD is emerging from cross-sectional studies using OCT. Reduced thickness in the ganglion cell and IPLs (GCIPL) was associated with poorer cognitive scores^18^ and with higher risk of developing PD dementia^12^; GCIPL was also thinner in patients with dementia with Lewy bodies (DLB) than idiopathic PD.^19^ Using cross-sectional modelling, retinal changes were linked with PD progression but were modelled to be a later event in the sequence of progression to dementia than higher order visual tests.^20^ However, few studies have examined structural retinal changes in PD longitudinally. One recent study found greater cognitive decline after 3 years in patients with PD, with thinnest retinal nerve fibre layer (RNFL) at baseline, compared with patients with thicker RNFL^21^ but did not examine other retinal layers. Another study found that reduced GCIPL and to a lesser degree, RNFL thickness, at baseline predicted an increased risk of cognitive decline at 3 years. Higher order visual measures were also predictive of cognitive decline.^19^ However, that study only examined relative risk of dementia, and not longitudinal differences in survival.

Therefore, despite prior work, key unanswered questions remain: (1) whether visual deficits in PD predominantly arise due to visual cognitive processing or retinal changes and (2) which of retinal or visual measures better predict longitudinal dementia-free survival in PD. Disambiguating these is critical to predict dementia more accurately and to provide insights into processes underlying PD dementia.

To address these questions, we examined visual function and retinal thickness in PD, with longitudinal assessment of cognition at 18-month and 36-month follow-up. We focused on the GCIPL, as phosphorylated alpha-synuclein has been found in the IPL at postmortem,^7^ and this layer has been most consistently linked with changes in PD.^9 12 22^ Given the widespread white matter changes seen with visuoperceptual deficits in PD, we hypothesised that patients with higher order visual changes would show greater conversion to dementia and poor outcomes over time. We further hypothesised that while retinal changes may relate to cognitive change over time, these associations would be weaker than for higher order visual deficits.

## Methods

### Participants

114 people with PD were recruited from our UK centre and affiliated clinics between October 2017 and November 2018. Inclusion criteria were a clinical diagnosis of PD using Queen Square Brain Bank Criteria,^23^ within 10 years of diagnosis, aged 49–80 years. 36 age-matched controls were recruited from unaffected spouses and university volunteer databases. Exclusion criteria were confounding neurological or psychiatric disorders, a diagnosis of dementia or Mini-Mental State Examination (MMSE) <25^24^ and ophthalmic disease sufficient to impair visual acuity (see Leyland *et al*).^12^ Ten participants were excluded due to ophthalmic disease (four PD/six controls). Participants gave written informed consent, and the study was approved by the Queen Square Research Ethics Committee (15.LO.0476).

### Clinical and ophthalmic evaluation

Participants underwent clinical and ophthalmic assessments at baseline; and clinical assessments after approximately 18 months (mean=16.01 months, SD=2.56) and 36 months (mean=38.96 months, SD=4.45). Participants were tested while on their usual medications and levodopa equivalent daily dose (LEDD) was calculated.^25^ Symptom severity was measured using the Movement Disorders Society Unified Parkinson’s disease Rating Scale (MDS-UPDRS).^26^ Participants were contacted for follow-up with researchers blind to visual performance and retinal status. A comprehensive baseline ophthalmic assessment, including a slit-lamp ophthalmic examination, and measurement of intraocular pressures using Goldmann applanation tonometry were performed by a consultant ophthalmologist.

### Assessments of visual function

Best-corrected visual acuity was measured binocularly using a LogMAR chart (with habitual lenses, if worn). Binocular contrast sensitivity was measured using a Pelli-Robson chart (SSV-281-PC) (http://www.sussex-vision.co.uk) at 1 m distance. Higher order visuoperception was measured using tasks probing distinct aspects of higher order visuoperception: the Cats-and-Dogs test and biological motion test. The Cats-and-Dogs test measures tolerance to visual skew,^27^which is reduced in PD and correlates with poorer cognitive function.^12 14 27^ Stimuli were generated using MATLAB as previously described^27^ (online supplemental methods). Images were presented centrally, subtending 4° × 13° of visual angle and shown for 280 ms, followed by a choice screen (response time 3800 ms), with three runs each of 90 repetitions (total time 15 min).

10.1136/jnnp-2023-331083.supp1 Supplementary data

Biological motion is the perception of a person moving when shown only point lights at the position of major joints.^28^ It has also been shown to be affected in PD,^29^ with worse performance relating to poorer cognition both cross-sectionally and longitudinally.^13^ Stimuli were generated using MATLAB as previously described^12^ (online supplemental methods). Stimuli consisted of point-light walkers (12 white dots on grey background, height 7^o^, 800 ms presentation time), with position and motion scrambled for control stimuli and motion-matched noise dots added adaptively to increase difficulty (225 repetitions, total time 15 min). We used the QUEST Bayesian adaptive method to calculate the number of additional noise dots tolerated for 82% accuracy.^30^ An overall measure of higher order visual function was calculated from the summed z-score of the Cats-and-Dogs and biological motion scores, calculated for each participant with reference to the entire group-average.

### Retinal structure: OCT

**Figure 1:**
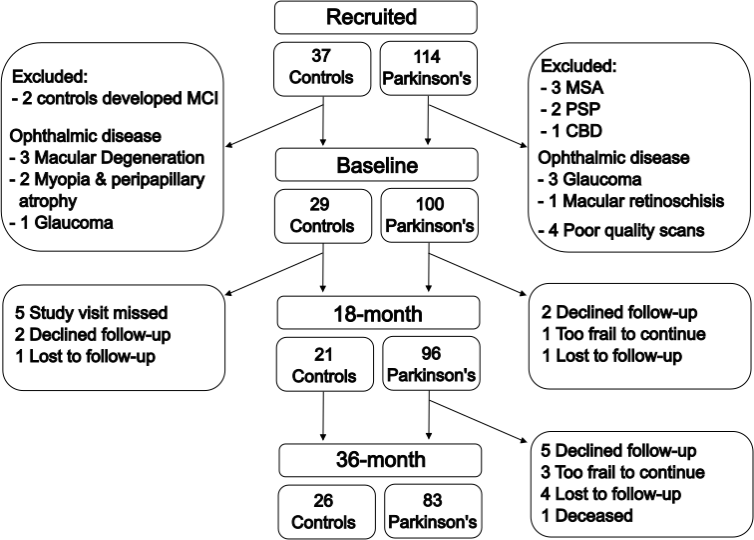
Patient flow in the study patients and controls recruited and tested in this study at baseline and during 18-month and 36-month follow-up visits. Due to the COVID-19 pandemic, the 18-month follow-up visits were missed for five controls. Note that patients who declined follow-up are not included in follow-up data but are logged as censored events for the time to survival analysis. CBD, cortical basal degeneration; MCI, mild cognitive impairment; MSA multiple system atrophy; PSP, Progressive supranuclear palsy.

Baseline retinal imaging was performed on both eyes. Retinal layer structure was measured using high-resolution spectral domain OCT (SD-OCT; Heidelberg HRA/Spectralis, Heidelberg Engineering, Heidelberg, Germany) in a dimly lit room after pharmacological mydriasis according to a standard protocol as previously described.^12^ Two identical OCT devices were used by four operators at one site (National Hospital for Neurology and Neurosurgery), using viewing module V.6.9.5.0. We used macular scan protocols in infrared-OCT mode (laser illumination for excitation: 486 nm), to acquire a simultaneous fundus image, with a reflectance wavelength of 816 nm. We acquired OCT scans with TruTrack eye-tracking to stabilise the retinal image. Macular thickness was measured using the volumetric macular protocol of the Spectralis SD-OCT device (NSite application) using an internal fixation source and centred on the fovea. The protocol consisted of 25 vertical line scans at a resolution of 1536 (scanning angle: 20°×20°, density: 240 µm, 4.7 scans/s, automatic real-time frames: 49). A separate scan of the optic disc was obtained using the standard ‘circle scan’ option within the NSite application. This measured peripapillary RNFL (pRNFL) thickness by projecting a 3.5 mm diameter scanning circle onto the retina at an angle of 12° nasal to fixation, thereby centred on the optic disc. This circle scan contains 768 A-scans along a peripapillary circle of 360°. We performed quality control of OCT data according to international consensus OSCAR IB standards^31^ and collected and reported OCT data according to international Advised Protocol for OCT Study Terminology and Elements (APOSTEL) guidelines.^32^ On this basis, four patients with PD were excluded due to poor-quality scans (see figure 1).

### OCT analysis

GCL, IPL and inner nerve layer (INL) as well as pRNFL thickness around the optic disc were computed using automatic layer segmentation, via Heidelberg software V.1.10.20, with the NSite module. Automatic segmentation was corrected manually for areas with failures by researchers blinded to participant status on visual and cognitive scores. Segmented layers were exported as a 6 mm Early Treatment Diabetic Retinopathy Study (EDTRS) grid^33^ and GCL and IPL thicknesses summed to provide a combined GCIPL total thickness. This was calculated for the macula and the 1 mm to 3 mm diameter ring area around the fovea (parafoveal GCIPL; pfGCIPL).

In patients with PD, the eye contralateral to the most symptomatic side, identified using the MDS-UPDRS-III, was selected for analysis. For controls and patients with symmetrical motor signs, the eye was selected randomly. For eight patients and one control who had pre-existing ophthalmic conditions in one eye, the other eye was included.

### Neuropsychological evaluation

Two tests per cognitive domain were assessed, in line with MDS guidelines.^34^ Cognitive assessments were conducted blind to visual-performance and retinal status. General cognitive function was assessed with the MMSE and Montreal Cognitive Assessment (MoCA). Memory was assessed with the Recognition Memory Test for words and immediate and delayed versions of the Logical Memory task. Language was assessed with the Graded Naming Test and letter fluency. Visuospatial abilities were tested with Benton’s Judgement of Line Orientation and the Hooper Visual Organisation Test. Executive functions were measured with the Stroop task from the Delis-Keplan Executive Function System and category fluency. Attention was tested using Stroop colour naming and Digit Span. For each cognitive task, a z-score was calculated with reference to the baseline performance of controls. A composite cognitive score was calculated as the averaged z-scores of the MoCA plus one task per cognitive domain (inverted Stroop colour naming time, category fluency, letter fluency, Recognition Memory Test and Hooper visual organisation test).

### Statistical analyses

Demographic, cognitive and visual measures and retinal thicknesses at baseline were compared between groups using two-tailed Welch’s t-tests or Mann-Whitney tests for non-normally distributed data. Repeated measures Analysis of Variance (ANOVAs) were used to compare visits within groups, using Greenhouse Geiser corrections where the assumption of sphericity was not met. Associations between visual measures and cognitive outcomes at each visit were tested using linear regression, adjusted for age.

To examine longitudinal progression of cognition, multivariable linear mixed-effect models (LMMs) were fitted using the lme4 package in R.^35^ The use of LMMs allowed the inclusion of participants with missing data at follow-up. Models were fitted separately in patients and controls, with fixed effects of baseline age, sex, time since baseline visit in years and a random intercept for participant to allow for repeated measures. Importantly, by including baseline as one of the timepoints for the cognitive outcome, the model enables us to account for baseline cognition. To test for effects of visual and retinal variables on cognitive change, an interaction term between time and retinal thickness or visual performance was added. Hypothesis testing for LMMs was performed using Satterthwaite’s approximation for *df* within the lmerTest package.^36^ The Akaike information criterion (AIC) and Bayesian information criterion (BIC) were used to compare the LMMs with higher order vision and retinal thickness as predictors.

Survival analyses were performed using the Kaplan-Meier method to calculate a non-parametric estimate of survival times and probabilities. These were calculated separately for dementia (defined as a reported dementia diagnosis or MoCA <26 and subsequently remaining <26); and for dementia plus death or frailty (defined as the participant becoming persistently too frail or unwell to attend research visits). Data were right censored for participants who withdrew or were lost to follow-up. The survival curves of low and high visual performers and of low and medium/high retinal tertiles were compared using log rank tests.

Retinal tertiles were defined based on a reference retinal thickness distribution of 250 controls aged 40–83 years, as previously described.^19^ The lowest tertile included participants with baseline pfGCIPL thickness below the 25th percentile (72.1–89.7 µm), participants within the IQR were classified as the medium tertile (89.7–98.9 µm) and the highest tertile included participants above the 75th percentile (98.9–116 µm). Visual performance groups in PD were defined as previously described^13^; participants scoring below the median on both the Cats-and-Dogs and biological motion tasks at baseline were defined as low-visual performers; all other participants as high-visual performers.

P<0.05, Bonferroni corrected for multiple comparisons (two comparisons for LMM and survival analyses, significance p<0.025; 14 comparisons for correlations with clinical measures, significance p<0.003; 25 comparisons for differences between clinical variables, significance p<0.002) was accepted as the threshold for statistical significance. Analyses were performed in R (R V.4.2.1; https://www.r-project.org/).

### Data availability

The data supporting the findings of this study are available from the corresponding author, on reasonable request.

## Results

### Demographics and clinical, visual and cognitive measures

During follow-up, six patients with PD were diagnosed with atypical Parkinsonism and two controls developed mild cognitive impairment (MCI). This resulted in a total of 100 people with PD who took part at baseline and is included in the analysis presented here (see figure 1). They were aged 49–82 years (mean 64.1 years, SD 7.95, 45 women (45%)), plus 29 unaffected controls (aged 50–80, mean 64.2 years, SD 8.56, 16 women (55%)) (figure 1).

Patients with PD and controls were well matched for age and gender, with no difference in overall cognition, visual acuity or retinal thickness. However, the PD group showed poorer visual cognition than controls (Hooper’s visual organisation test; W=1834, p=0.030) (table 1). Dividing patients with PD by higher order visual performance, 37 patients were classified as low-vision and 63 as high-vision performers. During follow-up, 14 participants developed dementia, 4 were too unwell to continue the study and two deaths occurred (one after cognitive follow-up). Additionally, seven patients discontinued the study for external reasons, and five were lost to follow-up (figure 1).

**Table 1:** Demographics and clinical variables

	Controls (n=29)	All PD (n=100)	Test statistic	P value	PD low vision (n=37)	PD high vision (n=63)	Test statistic	P value
Age, mean (SD)	64.2 (8.56)	64.1 (7.95)	W=1459	0.96	68.3 (7.70)	61.7 (7.03)	t=4.28	<0.0001**
Sex (F/M)	16/13	45/55	χ² = 0.57	0.45	14/23	31/32	χ² = 0.80	0.37
Disease duration (years), mean (SD)	–	4.14 (2.48)	–	–	4.70 (2.94)	3.81 (2.11)	W=1338	0.22
Age onset PD, mean (SD)	–	60.5 (8.08)	–	–	64.3 (8.75)	58.3 (6.81)	W=1613	0.001**
MDS-UPDRS total, mean (SD)	8.21 (5.12)	43.5 (20.6)	W=33	<0.0001**	46.5 (23.2)	41.7 (18.8)	W=1279	0.42
LEDD, mean (SD)	–	443 (259)	–	–	462 (239)	431 (271)	W=1274	0.44
Years of education, mean (SD)	17.4 (2.42)	17.2 (2.81)	W=1491	0.82	18.2 (2.61)	16.6 (2.77)	W=1512	0.013*
Cognitive measures								
MMSE, mean (SD)	29.1 (0.99)	28.9 (1.11)	W=1593	0.39	28.7 (1.15)	29.0 (1.08)	W=963	0.13
MOCA, mean (SD)	28.7 (1.37)	27.9 (2.05)	W=1774	0.061	27.0 (2.41)	28.4 (1.63)	W=757	0.003*
Composite Cognitive Score, mean (SD)	0.03 (0.61)	−0.29 (0.81)	W=1749	0.075	−0.67 (0.92)	−0.07 (0.65)	W=674	0.0008**
Word Recognition Task, mean (SD)	24.5 (1.09)	24.2 (1.10)	W=1798	0.029 *	23.7 (1.33)	24.4 (0.87)	W=815	0.007*
Logical Memory Immediate †, mean (SD)	15.7 (3.80)	15.3 (4.41)	t=0.40	0.70	14.8 (4.15)	15.6 (4.56)	t=0.77	0.44
Logical Memory Delayed †, mean (SD)	13.9 (3.95)	13.4 (4.19)	t=0.44	0.66	12.9 (4.18)	13.7 (4.21)	t=0.80	0.43
Graded Naming Task, mean (SD)	23.0 (6.17)	23.8 (2.96)	W=1629	0.31	23.1 (3.58)	24.1 (2.48)	W=1001	0.24
Verbal Fluency—letter, mean (SD)	16.6 (5.77)	16.7 (5.60)	W=1419	0.86	17.1 (6.85)	16.4 (4.76)	W=1218	0.71
Verbal Fluency—category, mean (SD)	22.4 (5.34)	21.7 (5.73)	t=0.67	0.51	20.1 (6.68)	22.6 (4.92)	t=1.98	0.053
Stroop Colour Naming time (s), mean (SD)	32.6 (7.73)	34.7 (7.79)	W=1218	0.22	36.6 (8.32)	33.6 (7.32)	W=1390	0.063
Stroop Word Reading time (s), mean (SD)	21.6 (5.47)	23.3 (4.83)	W=1033	0.026 *	23.5 (4.99)	23.2 (4.77)	W=1151	0.80
Stroop Interference time (s) mean (SD)	57.6 (13.6)	63.8 (20.7)	W=1235	0.25	70.4 (25.1)	60.0 (16.7)	W=1445	0.024 *
Hooper, mean (SD)	25.9 (2.04)	24.5 (3.15)	W=1834	0.030 *	22.8 (3.25)	25.5 (2.63)	W=575	**<**0.0001 **
Judgement of Line Orientation, mean (SD)	24.1 (5.87)	24.6 (3.78)	W=1449	0.94	23.3 (3.80)	25.3 (3.58)	W=767	0.007 *
Digit Span Forward †, mean (SD)	9.35 (2.06)	9.38 (1.88)	W=672	0.99	9.00 (2.07)	9.58 (1.74)	W=592	0.21
Digit Span Backward †, mean (SD)	7.00 (2.06)	7.20 (2.23)	W=628	0.68	7.11 (2.06)	7.25 (2.34)	W=723	0.93
Visual measures								
Acuity (LogMAR) ‡, mean (SD)	−0.11 (0.14)	−0.09 (0.13)	W=1274	0.32	−0.05 (0.14)	−0.11 (0.13)	W=1478	0.026 *
Contrast sensitivity (Pelli Robson)§, mean (SD)	1.84 (0.21)	1.79 (0.16)	W=1744	0.075	1.69 (0.15)	1.85 (0.14)	W=576	<0.0001 **
Higher-order vision: Cats-and-dogs, mean (SD)	2.14 (0.59)	1.90 (0.56)	t=1.93	0.060	1.44 (0.38)¶	2.17 (0.46)¶	t=8.66	<0.0001**
Higher order vision: biological motion, mean (SD)	15.7 (9.55)	16.6 (11.44)	W=1435	0.93	7.67 (4.07)¶	21.89 (11.10)¶	W=244	<0.0001**
Retinal thickness								
GCIPL—macular, mean (SD)	71.4 (5.74)	70.5 (6.07)	t=0.77	0.45	68.5 (6.26)	71.6 (5.70)	t=2.47	0.016*
GCIPL—parafoveal, mean (SD)	90.2 (7.19)	89.1 (7.81)	t=0.67	0.50	86.5 (8.37)	90.7 (7.07)	t=2.57	0.013*
RNFL—macular, mean (SD)	27.5 (2.69)	27.8 (3.08)	t=0.65	0.51	28.00 (3.12)	27.8 (3.07)	t=0.37	0.72
RNFL—peripapillary, mean (SD)	101.2 (12.8)	98.8 (13.2)	t=0.75	0.46	98.0 (9.98)	101.0 (13.47)	t=1.27	0.21
INL—macular, mean (SD)	32.6 (2.57)	32.8 (2.78)	t=0.31	0.76	32.3 (2.95)	33.1 (2.65)	t=1.39	0.17
INL—parafoveal, mean (SD)	37.2 (3.35)	38.0 (3.74)	t=1.15	0.26	37.1 (3.69)	38.6 (3.69)	t=1.88	0.064

*Show significant differences p<0.05. **Bonferroni corrected p<0.002.

†Due to a protocol change, obtained in only a proportion of participants, Controls n=17, All PD n=79, PD low vision n=28, PD high vision=51.

‡Lower scores on the LogMAR indicate better visual acuity (ie, improved visual performance).

§Higher scores on the Pelli Robson indicate better contrast sensitivity (ie, improved visual performance).

¶Low vision and high vision groups were split based on higher order vision tasks; hence we expect groups to differ on task performance.

GCIPL, ganglion cell layer and internal plexiform layer; INL, inner nerve layer; LEDD, levodopa daily equivalent dose; MDS-UPDRS, Movement Disorders Society Unified Parkinson’s Disease Rating Scale; MMSE, Mini Mental State Examination; MoCA, Montreal Cognitive Assessments; PD, Parkinson’s disease; RNFL, retinal nerve fibre layer.

Patients with poor higher order vision at baseline were older, with a later disease onset and had poorer global cognition (MoCA). They completed more years of education and had poorer visual acuity than those with intact higher order visual functioning. GCIPL was also thinner in patients with worse higher order vision (GCIPL: t=2.47, p=0.016; pfGCIPL: *t*=2.57, p=0.013), with no differences in RNFL (RNFL: *t*=0.37, p=0.72; pRNFL: *t*=1.27, p=0.21) or INL (INL: t=1.39, p=0.17; pfINL, t=1.88, p=0.064) (table 1).

When dividing patients according to retinal thickness tertiles, 53 patients were in the low-pfGCIPL and 48 in the medium/high-pfGCIPL thickness group. Patients in the lowest pfGCIPL thickness tertile were older, with later age of onset versus the medium/high thickness group, and a trend towards having a lower MoCA score. Higher order visual function was poorer in patients with low retinal thickness (online supplemental table 1). As expected, across three visits, the PD group showed increased requirement for dopaminergic agents (LEDD) and overall symptoms (MDS-UPDRS total). Likewise, visual acuity and contrast sensitivity worsened over time (online supplemental table 2).

10.1136/jnnp-2023-331083.supp2 Supplementary data

### Relationship between visual measures and cognition

**Figure 2:**
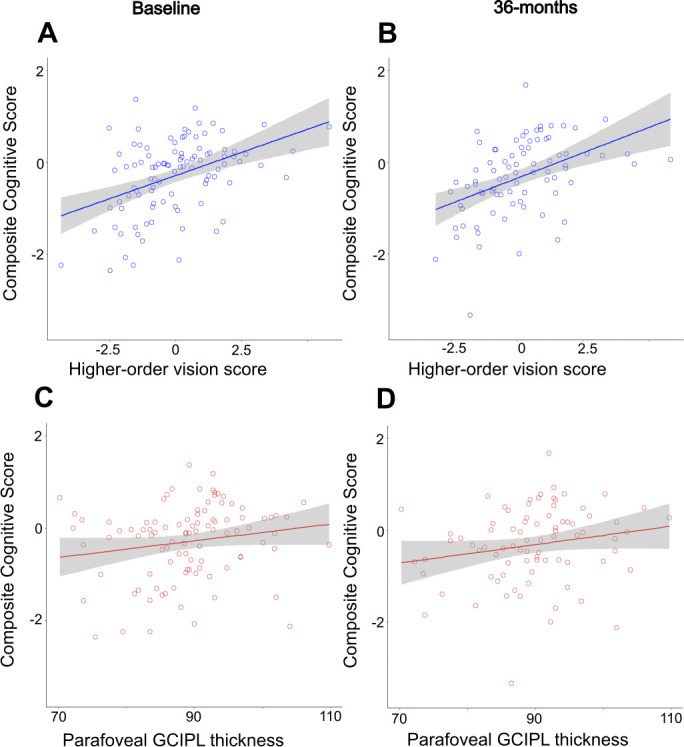
Relationship between visual function and cognitive measures at (A) baseline and (B) after 36-month follow-up; and between GCIPL retinal thickness and cognitive measures at (C) baseline and (D) after 36-month follow-up. Scatter plots display original data, without adjustment for age. GCIPL, ganglion cell—inner plexiform layer; MoCA, Montreal Cognitive Test. Shaded area represents 95% CIs.

Within PD, better baseline higher order vision was associated with better composite cognition at baseline: for a 1-point increase in the higher order vision score, baseline-combined cognition increased by 0.16, adjusted for age (β=0.16, SE=0.05, p=0.003). Better baseline higher order vision was also associated with higher scores at both follow-ups (18 months: β=0.20, SE=0.05, p=0.0009; 36 months: β=0.22, SE=0.06, p=0.0003), adjusted for age (table 2, figure 2). Relationship between higher order vision and MoCA was at trend at baseline (β=0.27, SE=0.14, p=0.051), but there was a significant association between baseline visual performance and MoCA after 36 months (β=0.39, SE=0.17, p=0.021). No associations were observed between cognition and other visual measures such as visual acuity and retinal thickness, with the exception of contrast sensitivity, which was positively associated with both baseline MoCA (β=3.72, SE=1.35, p=0.007) and composite cognitive score (β=1.38, SE=0.53, p=0.011) in the PD group but not at 36-month follow-up (MOCA, β=−0.25, SE=1.77, p=0.89; composite cognitive score, β=0.79, SE=0.64, p=0.23) (table 2).

**Table 2:** Relationship between baseline visual measures and cognitive scores in the Parkinson’s disease group at each visit, adjusted for age

Baseline visual measure	Baseline cognitive composite (n=99)			18-month cognitive composite (n=92)			36-month cognitive composite (n=82)		
	β	SE	P	β	SE	P	β	SE	P
LogMAR (acuity)	−0.65	0.62	0.29	0.10	0.68	0.89	0.10	0.68	0.86
Pelli Robson (Contrast Sensitivity)	1.38	0.53	0.011*	0.84	0.61	0.17	0.79	0.64	0.23
Higher-order vision	0.16	0.05	0.003 **	0.20	0.05	0.0009**	0.22	0.06	0.0003**
GCIPL—macular	0.004	0.01	0.73	0.006	0.01	0.67	0.01	0.02	0.37
GCIPL—parafoveal	0.005	0.01	0.61	0.003	0.01	0.78	0.01	0.01	0.23
INL—parafoveal	−0.01	0.02	0.57	−0.007	0.02	0.77	0.04	0.03	0.089
RNFL—peripapillary	0.003	0.006	0.66	−0.004	0.007	0.61	−0.008	0.007	0.25
**Baseline Visual Measure**	**Baseline MoCA (n=100)**			**18-month MoCA (n=95)**			**36-month MoCA (n=83)**		
	**β**	**SE**	**P**	**β**	**SE**	**P**	**β**	**SE**	**P**
LogMAR (acuity)	−1.17	1.60	0.46	3.50	2.21	0.12	−1.06	2.01	0.60
Pelli Robson (Contrast Sensitivity)	3.72	1.35	0.007*	4.08	1.94	0.039*	−0.25	1.77	0.89
Higher-order Vision	0.27	0.14	0.051	0.30	0.20	0.14	0.39	0.17	0.021*
GCIPL—macular	0.01	0.03	0.67	−0.02	0.05	0.62	0.05	0.04	0.26
GCIPL—parafoveal	0.004	0.03	0.87	−0.03	0.04	0.39	0.04	0.03	0.27
INL—parafoveal	0.003	0.05	0.99	0.0051	0.08	0.95	0.12	0.07	0.078
RNFL—peripapillary	0.02	0.02	0.32	0.005	0.03	0.84	−0.01	0.02	0.62

β: Difference in mean cognitive score for a one-unit change in predictor variable, adjusted for age.

*Show significant differences p<0.05, ** Bonferroni corrected p<0.003.

GCIPL, ganglion cell layer and internal plexiform layer; INL, inner nerve layer; MoCA, Montreal Cognitive Assessment; RNFL, retinal nerve fibre layer.

### Predictive value of higher order visual function and retinal thickness for cognitive outcomes in Parkinson’s disease

**Figure 3:**
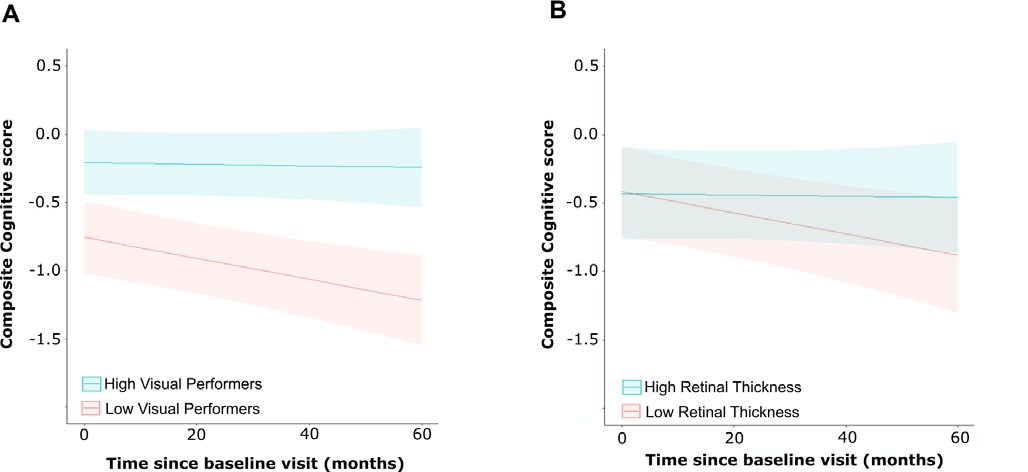
Risk of cognitive decline predicted by higher order visual measures and retinal thickness. Modelled data for predicted composite cognitive score over time in the Parkinson’s disease group, based on (A) baseline higher-order visual function z-scores of +1.5 (high visual performer) and −1.5 (low visual performer); (B) baseline parafoveal GCIPL thickness z-scores of +1.5 (high retinal thickness) and −1.5 (low retinal thickness). Estimated marginal means from linear mixed effect models were calculated, and the lines plotted holding age and gender constant. Shaded area represents 95% CIs. GCIPL, ganglion cell layer and internal plexiform layer.

As expected, patients with PD showed a decline in cognitive composite score over time of −0.039 units per year (table 3). Controls showed a smaller and non-significant decline over time, although there was not a significant difference in the decline over time between groups (p=0.70 interaction test). Importantly, in PD, worse higher order visual scores significantly predicted lower cognition at baseline and a greater decrease in cognitive score over time, when accounting for baseline cognition. For a one-unit decrease in the baseline higher order visual score, the combined cognitive score was predicted to decrease by 0.178 (β=0.178, SE=0.050, p=0.0005) and the decrease per year in cognitive score to be 0.024 points greater (β=0.024, SE=0.001, p=0.013) (table 3, figure 3A). In contrast, retinal thickness was not predictive of differences in the cognitive composite scores at baseline or the change in cognitive score over time in PD (pfGCIPL, β=−0.013, SE=0.080, p=0.87; pfGCIPL* time interaction, β=0.024, SE=0.001, p=0.12;) (table 3, figure 3B). Both the AIC and BIC of the higher order vision model (AIC=504.30, BIC=533.14) showed better fit compared with the pfGCIPL thickness model (AIC=525.20, BIC=554.05) in a non-nested model comparison, consistent with higher order vision better predicting PD dementia than retinal thickness. When excluding participants with hypertension and/or diabetes, these results were still observed (online supplemental table 3).

**Table 3:** Linear mixed-effect model effects estimate of longitudinal combined cognitive score

Model	Controls			Parkinson’s Disease		
	β	SE	P	β	SE	P
Simple						
Time from baseline	−0.029	0.002	0.15	−0.039	0.001	0.018**
Baseline age	−0.009	0.014	0.52	−0.048	0.009	0.0001**
Retinal						
Time from baseline	−0.029	0.002	0.15	−0.045	0.001	0.011**
pf GCIPL	0.020	0.134	0.88	−0.013	0.080	0.87
pf GCIPL * time	0.006	0.002	0.79	0.024	0.001	0.12
Baseline age	−0.009	0.014	0.53	−0.036	0.010	0.0006**
Vision						
Time from baseline	−0.037	0.002	0.083	−0.048	0.001	0.006**
Higher order vision	−0.025	0.081	0.76	0.178	0.050	0.0005**
Higher order vision * time	−0.029	0.001	0.035*	0.024	0.001	0.013**
Baseline age	−0.008	0.014	0.58	−0.011	0.010	0.27

Higher order vision: as measured using computerised tasks: Cats and dogs and biological motion.

β: difference in cognitive z-score for a one-unit increase in predictor. For time from baseline, this represents the rate of change per year in cognitive z-score, for interactions with time, this represents the effect of a one-unit increase in the variable on the rate of change in the cognitive z-score.

*Show significant differences p<0.05, **Bonferroni corrected p<0.025.

pfGCIPL, parafoveal ganglion cell layer and internal plexiform layer.

### Prediction of dementia-free survival

**Figure 4:**
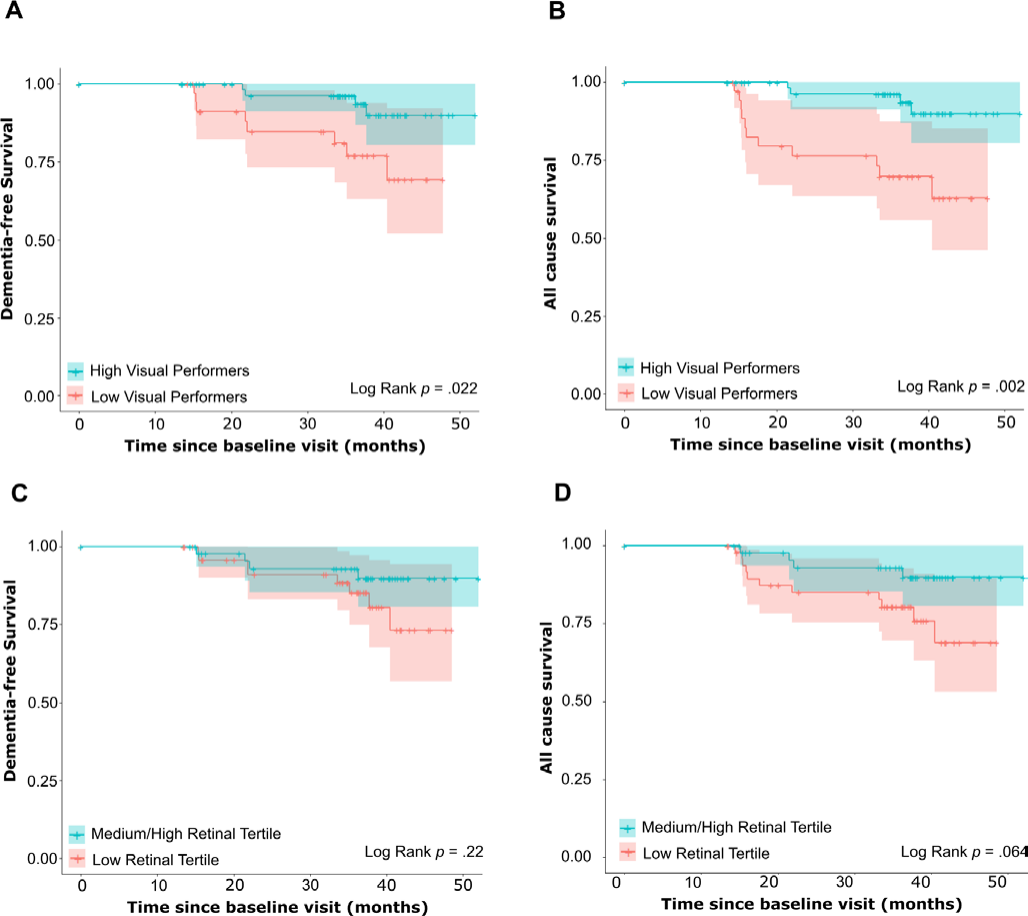
Survival curves for prediction of dementia, death and frailty in Parkinson’s disease Kaplan-Meier plots illustrating the probability of remaining free from (A) dementia and (B) cumulative death, dementia, or frailty in low versus high visual performers. Low visual performers scored below the median on two tests of higher-order visual perception, as in our previous work.^13^ Kaplan-Meier plots illustrating the probability of remaining free from (C) dementia and (D) cumulative death, dementia, or frailty in medium/high versus low retinal tertiles. Retinal tertiles were based on the parafoveal GCIPL thickness of a reference retinal thickness distribution (as in previous work.^19^ Dementia was defined using clinical diagnosis or MMSE score <26. Frailty was coded as participants/carers reporting being unable to attend research visits due to frailty and was assessed blind to visual or retinal status. Shaded area represents 95% CIs. GCIPL, ganglion cell layer and internal plexiform layer; MMSE, Mini Mental State Examination.

Time-to-event analysis found that, when examining higher-order visual performance, those patients with low visual performance at baseline had an increased rate of dementia after follow-up (χ² (1) = 5.2, p=0.022); and of dementia, death or frailty (χ² (1) = 10.0, p=0.002) compared with those patients who showed high visual performance (figure 4A,B). Comparison of the medium/higher and low pfGCIPL tertiles found no association of retinal tertile with risk of dementia (χ² (1)=1.5, p=0.22) and a trend towards an association with combined risk of dementia, death or frailty (χ²(1)=3.4, p=0.064) (figure 4C,D).

## Discussion

In this large longitudinal cohort, higher order visual function, but not retinal structure, predicted cognitive decline and poor outcomes in PD. Specifically, poor higher order vision at baseline was associated with worsening cognition and increased probability of death, dementia or frailty over 3 years. Conversely, baseline GCIPL thickness did not predict cognition or adverse outcomes. Our findings provide evidence that higher order visual changes in PD show greater predictive value over retinal thickness for incipient dementia and provide insights into the sequence of degenerative changes in PD, with cortical neurodegeneration potentially an earlier event than retinal degeneration. Our finding that higher order vision predicts outcomes in PD is consistent with previous work relating visual performance with future cognitive decline^1 11 13^ and that visual function in general relates to dementia and poor outcomes in PD.^15^

While OCT-derived measures have consistently shown GCIPL thinning in patients with PD versus controls,^9 37^ the temporal relationship between GCIPL thickness and cognitive decline is less clear. We have previously shown cross-sectionally that GCIPL thickness relates to dementia risk, estimated from clinical algorithms, in PD.^12^ Using cross-sectional data and computational modelling, we estimated that retinal changes in PD are likely to occur after higher order visual dysfunction and structural brain changes.^20^ Other cross-sectional studies have suggested a relationship between GCIPL and poorer cognition in PD.^18 38^ However, we found no relationship between baseline GCIPL thickness and cognition in our cohort. Notably, where this relationship has been seen, cognition was lower in PD (eg, mean MoCA=23.9^18^; mean MMSE=26,^38^ compared with a baseline MoCA of 27.9 in our cohort. An association between cognition and retinal thickness may be more evident with a greater range of cognitive impairment; or at later stages.

Few studies have investigated the longitudinal relationship between retinal thickness and cognition. One study examined RNFL in PD, with 3 years’ longitudinal cognitive evaluation.^21^ Using age-adjusted LMMs, they found a greater decline in MMSE with increasing disease duration for patients with thinner baseline RNFL, although they did not show a direct relationship with time. Another study examined higher order visual function and retinal thickness in PD and DLB.^19^ In addition to a relationship between GCIPL and MoCA at baseline, the authors reported a relationship between thinner RNFL, GCIPL and higher order visual function with greater cognitive decline after 3 years. However, the PD group had impaired baseline cognition (mean MOCA=24), suggesting significant cognitive deficits. They also included a group carrying severe *GBA* mutations, an extreme phenotype conferring a high dementia risk, who showed grossly impaired cognitive (mean MoCA=19), visuospatial and motor function. This may have driven some findings in that work. When examining cognition, that study found increased risk of cognitive decline for idiopathic patients with PD with thinnest GCIPL, although it is unclear whether age was adjusted for. They found an increased relative risk for cognitive decline in patients with poorer higher order visual function at baseline, which did not survive adjustment for confounders. Although LMMs were performed, it is unclear whether retinal or visual performance predicted cognitive decline in the overall model.

In contrast, our study included patients with no baseline cognitive involvement and examined the effects of higher-order visual function and retinal structure on cognitive decline. Using LMMs adjusted for age, we showed that higher order visual dysfunction significantly predicted longitudinal cognitive decline, but that retinal thickness showed no predictive effects for cognition. Consistent with these findings, our survival analyses showed a greater conversion to dementia, death or frailty in patients with poorer baseline higher order visual function, but not for those with thinner retinas. Importantly, our mixed model analysis allowed us to specifically take into account baseline cognition, showing that these tests provide additional information to standard cognitive tasks and confirm the utility of these or similar higher-order visual tests as stratification tools for the clinic or in clinical trials.

Two competing theories could explain retinal and higher order visual dysfunction in PD: one model suggests that degenerative changes arise in cortical regions and spread to the retina through a process such as retrograde trans-synaptic axonal degeneration; the other proposes that both cortical and retinal cells are vulnerable to degeneration in PD, and that de-arborisation occurs idiosyncratically throughout the nervous system, which might affect retina and cortex at similar times when examining a population.^39^ Our prospective study supports the former model, with primary neurodegeneration likely affecting cortical regions before involving distal areas such as the retina.

The higher order visual tests in our study used a computerised platform with carefully controlled visual stimuli and multiple trials. However, in an exploratory analysis, a simple visual cognition test also predicted cognition in PD (online supplemental table 4). The Hooper visual organisation test is quick to perform, needs minimal training and no special equipment. Simple visual cognition tests could be used in clinical or trial settings to aid prognostication and patient stratification. This will be relevant for trials of emerging disease-modifying treatments where selecting a more rapidly progressive cohort could improve trial efficiency.

### Limitations and future work

Patients with PD with poorer higher order vision were older than those with intact vision. The relationship between age and poorer vision is well-established,^40^ and older disease onset is consistently associated with a higher risk of dementia in PD.^41^ We corrected for age in our LMMs and demonstrated predictive effects of visual dysfunction independent of age.

During the follow-up period, six patients with PD and two controls received diagnoses of atypical Parkinsonism and MCI and were excluded from further analyses. These rates are in-line with other published longitudinal PD cohorts.^42^

We examined retinal structure using SD-OCT, however, functional measures of retinal change such as electroretinography may have greater sensitivity to predict outcomes in PD.^43^

For future extensions of this work, longer follow-up duration will be important to further establish the relationship between visual dysfunction, retinal structure and progression to dementia in PD. It will be helpful to validate whether simple and accessible forms of visual testing, in larger populations, can robustly predict cognitive change. Finally, pathological confirmation of cortical versus retinal involvement in a prospective cohort would provide definitive evidence for underlying processes driving neurodegeneration in PD.

### Summary

In a prospective cohort of patients with PD, we showed that visual function, but not retinal thickness, predicts cognitive decline and poorer outcomes over 3 years. Our work provides mechanistic insights into the likely sequence of changes underlying neurodegeneration in PD dementia with earlier higher order visual changes; and shows that visual tests, rather than retinal thickness measures, are likely to be effective and useful for patient stratification in clinical trials.

## Data Availability

Data are available upon reasonable request. The data supporting the findings of this study are available from the corresponding author, upon reasonable request.
