# Supplementary material for: Visual dysfunction is a better predictor than retinal thickness for dementia in Parkinson’s disease
Source: J Neurol Neurosurg Psychiatry. 2023 Apr 20;94(9):742–50. doi: 10.1136/jnnp-2023-331083 (PMC10447370; doi:10.1136/jnnp-2023-331083)
Supplement: Supplementary data [file jnnp-2023-331083supp001.pdf]

Supplementary Methods

Assessments of Visual Function

For the Cats-and-Dogs test, 1000 images of cats and dogs were obtained from an open online database (www.kaggle.com), cropped to equal size (300 x 300 pixels), converted to greyscale, and Fourier transforms of each image were computed. The average magnitude matrix of all images was stored for the run, and combined with the phase matrix of a single cat or dog image; plus a constant proportion of white noise (0.5). In this way, low-level image statistics were kept constant across the images of each run. The image was then skewed by a variable amount of skew along the x-axis, using 11 different levels of skew, and with direction of skew always to the left. Pseudo-random presentation was used to avoid participants gradually tilting their head toward the direction of skew.

To calculate discrimination sensitivity, missing trials were excluded and performance at each level of skew calculated. A sigmoid psychometric curve was fitted and threshold for image detection was calculated at 75% performance. For participants where a sigmoid curve could not be fitted (n=6), a threshold was estimated using that individual's overall performance (%) and the group's linear relationship between overall performance (%) and fitted threshold.

For biological motion stimuli consisted of point-light walkers (12 white dots on grey background, height 7°, 800ms presentation time), with position and motion scrambled for control stimuli; and motion-matched noise dots added adaptively to increase difficulty (225 repetitions, total time 15 minutes). Stimuli were generated within MATLAB Psychophysics Toolbox 3 and presented on a Dell Latitude 3340 in a darkened room

Supplementary Results

Regression Model: baseline higher-order vision and baseline cognitive score

As a further test of whether higher order visual tests add predictive value beyond the standard cognitive tests, a linear regression was run. Baseline cognitive score, baseline higher-order visual function and baseline age were included as predictors of cognition at 36-month follow up. We found that each of cognition and higher-order vision were significant predictors. Specifically, we found that baseline higher-order vision ($\beta = 0.13$, $t = 3.07$, $p = .003$) and baseline cognition ($\beta = 0.77$, $t = 9.10$, $p < .0001$) were each significant predictors of cognition after 36-months, even when both were accounted for in the model.
