# Supplementary material for: Visual dysfunction is a better predictor than retinal thickness for dementia in Parkinson’s disease
Source: J Neurol Neurosurg Psychiatry. 2023 Apr 20;94(9):742–50. doi: 10.1136/jnnp-2023-331083 (PMC10447370; doi:10.1136/jnnp-2023-331083)
Supplement: Supplementary data [file jnnp-2023-331083supp002.pdf]

Supplementary Table 1. Demographics and clinical variables for retinal tertile groups.

	Low retinal tertile (n = 52)	Medium/high retinal tertile (n= 48)	Test statistic	*p* Value
Age	66.40 (8.29)	61.65 (6.83)	t = 3.14	.002 **
Sex (F/M)	21 / 31	24 / 24	χ^2 = 0.58	.44
Disease Duration	4.23 (2.34)	4.04 (2.64)	W = 1323	.61
Age Onset PD	62.6 (8.72)	58.2 (6.71)	W = 1598	.016 *
MDS-UPDRS Total	44.1 (20.8)	42.8 (20.5)	W = 1291	.77
LEDD	467 (275)	416 (240)	W = 1386	.34
Years of Education	17.1 (2.87)	17.3 (2.39)	W = 1179	.63
**Cognitive Measures**				
MMSE, mean (SD)	28.8 (1.09)	29.1 (1.13)	W = 1012	.087
MOCA, mean (SD)	27.5 (2.31)	28.3 (1.65	W = 997.5	.077
Composite Cognitive Score, mean (SD)	-0.49 (0.82)	-0.08 (0.74)	W = 806	.003*
Word Recognition Task, mean (SD)	24.0 (1.16)	24.3 (1.022)	W = 1009	.074
Logical Memory Immediate [a], mean (SD)	15.4 (4.77)	15.1 (4.02)	t = 0.30	.77
Logical Memory Delayed [a], mean (SD)	13.8 (4.76)	13.0 (3.48)	t = 0.83	.41
Graded Naming Task, mean (SD)	23.7 (3.08)	23.9 (2.86)	W = 1225	.87
Verbal Fluency - letter, mean (SD)	15.6 (5.78)	17.9 (5.20)	W = 927	.026 *
Verbal Fluency - category, mean (SD)	21.0 (6.25)	22.4 (5.08)	t = 1.19	.24
Stroop Colour Naming time (s) , mean (SD)	35.5 (7.83)	33.9 (7.73)	W = 1405	.21
Stroop Word Reading time (s) , mean (SD)	23.5 (4.88)	23.1 (4.81)	W = 1253	.70
Stroop Interference time (s) , mean (SD)	64.6 (20.5)	62.9 (21.0)	W = 1337	.43
Hooper, mean (SD)	23.7 (3.02	25.3 (3.12)	W = 863	.007 *
Judgement of Line Orientation, mean (SD)	23.5 (3.60)	25.7 (3.65)	W = 773	.001 **
Digit Span Forward [a], mean (SD)	9.26 (1.85)	9.51 (1.92)	W = 704	.47
Digit Span Backward [a], mean (SD)	6.81 (2.27)	7.65 (2.14)	W = 612	.10
**Visual Measures**				
Acuity (LogMAR) [b], mean (SD)	-0.06 (0.14)	-0.11 (0.12)	W = 1502	.079
Contrast sensitivity (Pelli Robson) [c], mean (SD)	1.76 (0.18)	1.83 (0.14)	W = 1018	.088
Higher-order vision: Cats-and-dogs, mean (SD)	14.2 (9.80)	19.3 (12.56)	W = 942.5	.035 *
Higher-order vision: biological motion, mean (SD)	1.78 (0.59)	2.03 (0.50)	t = 2.29	.024 *
**Retinal thickness**				
GCIPL – macular [d], mean (SD)	66.3 (3.90)	75.0 (4.53)	t = 10.29	<.0001 **
GCIPL – parafoveal [d], mean (SD)	83.5 (5.31)	95.2 (4.89)	t = 11.55	<.0001 **

RNFL – macular [d], mean (SD)	27.1 (3.09)	28.7 (2.88)	t = 1.61	.010 *
RNFL – peripapillary [d], mean (SD)	94.7 (10.18)	105.4 (12.11)	t = 4.77	<.0001 **
INL – macular [d], mean (SD)	32.1 (2.35)	33.6 (3.00)	t = 2.89	.004 *
INL - parafoveal [d], mean (SD)	37.1 (3.25)	39/1 (3.96)	t = 2.82	.006 *

GCIPL= Ganglion Cell Layer and Internal Plexiform Layer, INL = Inner Nerve Layer. LEDD= Levodopa daily equivalent dose. MDS-UPDRS = Movement Disorders Society Unified Parkinson's Disease Rating Scale. MMSE = Mini Mental State Examination. MoCA = Montreal Cognitive Assessments. PD = Parkinson's Disease. RNFL= Retinal Nerve Fibre Layer.

* Show significant differences p<.05, ** Bonferroni corrected p<.002

[a] Due to a protocol change, Low Retinal Tertile n = 42, High Retinal Tertile = 37.

[b] Lower scores on the LogMAR indicate better visual acuity (i.e., improved visual performance).

[c] Higher scores on the Pelli Robson indicate better contrast sensitivity (i.e., improved visual performance).

[d] Retinal tertile groups were split based on GCIPL thickness tasks; hence we expect groups to differ on measures of retinal thickness.

Supplementary Table 2. Clinical variables for Parkinson's disease group at each visit.

	**Baseline (n = 100)**	**18-months (n = 96)**	**36-months (n = 83)**	***F*** [a]	***p*** [a]
MDS-UPDRS Total	43.5 (20.6)	38.9(22.5)	53.9(17.6)	20.83	<.0001**
MoCA	28.7 (1.37)	27.6 (2.88)	28.0 (2.27)	0.76	.47
LEDD	443 (259)	564 (343)	691 (350)	37.15	<.0001**
Acuity (LogMAR) [b]	-0.09 (0.13)	-0.02 (0.16)	0.03 (0.14)	27.8	<.0001 **
Contrast sensitivity (Pelli Robson) [c]	1.79 (0.16)	1.77 (0.17)	1.71 (0.16)	15.7	<.0001 **

LEDD= Levodopa daily equivalent dose. MDS-UPDRS = Movement Disorders Society Unified Parkinson's Disease Rating Scale. MoCA = Montreal Cognitive Assessments.

* Show significant differences p<.05, ** Bonferroni corrected p<.002

[a]Test statistic and *p* value from ANOVA test – this includes only participants who completed all 3 visits (n = 83)

[b] Lower scores on the LogMAR indicate better visual acuity (i.e., improved visual performance).

[c] Higher scores on the Pelli Robson indicate better contrast sensitivity (i.e., improved visual performance).

Supplementary Table 3. Linear Mixed-effect Model parameters; excluding participants who have Diabetes and/or Hypertension

	Controls			PD		
**Model**	**Estimate**	**SE**	***p***	**Estimate**	**SE**	***p***
**Simple**						
Time from baseline	-0.024	0.002	.42	-0.048	0.002	.026*
Baseline Age	-0.009	0.015	.55	-0.049	0.012	<.0001 **
**Retinal**						
Time from baseline	-0.024	0.002	.44	-0.056	0.002	.016**
pGCIPL	0.109	0.200	.59	0.0003	0.101	.99
pGCIPL * time	-0.034	0.003	.31	0.034	0.002	.16
Baseline Age	-0.008	0.016	.62	-0.047	0.012	.0003 **
**Vision**						
Time from baseline	-0.024	0.002	.34	-0.056	0.002	.015**
Higher-order vision	-0.043	0.099	.67	0.199	0.059	.001 **
Higher-order vision * time	-0.024	0.001	.26	0.024	0.001	.024 **
Baseline Age	-0.011	0.018	.54	-0.020	0.012	.11

PD = Parkinson's Disease. pGCIPL= Parafoveal Ganglion Cell Layer and Internal Plexiform Layer,

* Show significant differences p<.05, ** Bonferroni corrected p<.025

Supplementary Table 4. Linear Mixed-effect Model parameters, with Hooper visual organisation test as a predictor.

	Controls			PD		
**Model: Hooper**	**Estimate**	**SE**	***p***	**Estimate**	**SE**	***p***
Time from baseline	-0.43	0.02	.10	0.17	0.01	.22
Hooper	0.13	0.05	.008 **	0.14	0.02	<.0001**
Hooper * time	0.015	0.001	.13	0.006	0.0004	.33
Baseline Age	-0.002	0.01	.88	0.02	0.01	.051

PD = Parkinson's Disease

* Show significant differences p<.05, ** Bonferroni corrected p<.025
